# Health Rules in Time of Cholera

**Published:** 1888-04

**Authors:** 


### PERSONAL HEALTH-RULES IN TIME OF CHOLERA.

Sanitätsrath Dr. Paul Sachse, of Berlin, acting on what he believes to be a well-grounded supposition that cholera is due to the cholera bacillus, has made out a set of health-rules, to be freely distributed in time of cholera, so that "everyone can carry a copy in his pocket, and hang one on his mirror, or on the wall like a calendar." The rules are as follows :

Cholera is caused by infection by the microscopic organism called the comma-bacillus, on account of its peculiar form in cholera. These get into the human intestine, increase rapidly under favorable circumstances, and cause the peculiar symptoms of cholera, which always begins with an apparently harmless diarrhœa, that continues for several hours before the disease breaks out with force and becomes dangerous to life.

The possibility of infecting one's self in time of cholera with the bacillus is increased a thousand fold by assemblages and intercommunication of people. The outbreak of the disease is favored by anything that causes any stomach or intestinal trouble.

Since we have no absolutely certain means of controlling cholera after it has broken out, we should especially beware of becoming infected, and should take all precautions to kill, or at least to render as harmless as possible, the cholera germ before it gets into our bodies ; and by a regular mode of living and prudent deportment avoid anything that can disorder the digestive apparatus.

Since the cholera germ gets into the stomach and intestines by way of the mouth, we should take care :

1. To take only cooked food and drink. This is the most important rule. Even the water used for washing, rinsing, and bathing should be free from germs, and in cities water from wells should never be used, but only that from the city water-pipes.

2. To keep the body clean, and especially the hands, by frequent washing, especially before meals ; and this should be done with disinfecting solutions, such as a 5 per cent. solution of carbolic acid (or a $\frac{1}{8}$ per cent. solution of sublimate), and of this in time of cholera at least a quart should be used for washing the hands.

3. To live judiciously and carefully in time of cholera; and

(a) Not run away.

(b) Not to harbor people from cholera places.

(c) Not to visit a house in which there is cholera.

(d) Still less to eat or drink anything in such a house.

(e) Especially to take nothing, food, linen, laundry, playthings, or anything else, from houses in which there is cholera.

(f) To avoid in every way anything that may disturb digestion. Therefore :

Avoid taking cold, and sudden cooling off after being heated.

Do not sit up late at night with friends (drinking cold beer, for example).

Do not wear clothing that is very thin, and do not take off underclothing suddenly.

On no account bathe in running water.

Water-courses often bear the germs of cholera.

Avoid collections of people, fairs, festivals, etc., of all kinds.

4. All kinds of food are to be avoided that may cause catarrh of the stomach and intestines ; so also, over-eating and over-drinking are to be avoided.

What may one eat and drink ? What is forbidden ? What allowed ?

| FORBIDDEN ! ! | ALLOWED. |
|---|---|
| Unboiled water. | Boiled water, with or without cognac, arrac, or red wine. |
| Raw milk, raw cream, sour milk, and whipped cream. | Good soda or seltzer water, and natural mineral waters. |
| Butter and buttermilk. | Red wine. |
| Fresh baked bread. | Good lager beer. |
| All cold soups. | Coffee, tea, cocoa. |
| Cold cut meat that has stood for a long time. | Baked bread must be heated for one-half hour before being used. |
| All kinds of salad. | All well-cooked soups. |
| Mayonnaises and crêmes. | All hot (boiled, roasted, or stewed) meats. |
| Raw fruit, and unfermented fruit-juices. | All kinds of cooked vegetables (potatoes, rice, asparagus, green pease, cauliflower), and macaroni. |
| Cheese. | Fresh-cooked, warm compôtes. |
| Cookies. | Eggs, and egg preparations. |
| Ice. | Puddings. |

GOOD DAILY DIET.

*Early Breakfast.*—Coffee, tea, or cocoa (with or without well-boiled milk), eggs, bread that has been well heated and dried in an oven or stove for one-half hour (bread that has been cooked a second time) *without* butter.

*Breakfast.*—Bouillon with egg, bread as above, warm meat, wine.

*Noon meal.*—Hot soup, boiled, stewed, or roast meat, vegetables, fresh cooked compôte, red wine, or good beer.

*Tea.*—Coffee or tea.

*Supper.*—Tea, or soup made from the meat left over at the noon meal, with the morning's bread, or warm meat. Wine or beer as above.

5. Every irregularity of the body should be most strictly guarded against in time of cholera. Apparently slight diarrhœa should on no account be neglected, but a physician should be consulted at once.

